# Sea urchin growth dynamics at microstructural length scale revealed by Mn-labeling and cathodoluminescence imaging

**DOI:** 10.1186/s12983-017-0227-8

**Published:** 2017-08-25

**Authors:** Przemysław Gorzelak, Aurélie Dery, Philippe Dubois, Jarosław Stolarski

**Affiliations:** 1 Institute of Paleobiology, Polish Academy of Sciences, Twarda 51/55, 00-818 Warsaw, Poland; 2 Laboratoire de Biologie marine, Faculté des Sciences, Université Libre de Bruxelles, CP 160/15, av., F.D.Roosevelt, 50, B-1050 Bruxelles, Belgium

**Keywords:** Biomineralization, Calcite, Labeling, CL, Manganese

## Abstract

**Background:**

Fluorochrome staining is among the most widely used techniques to study growth dynamics of echinoderms. However, it fails to detect fine-scale increments because produced marks are commonly diffusely distributed within the skeleton. In this paper we investigated the potential of trace element (manganese) labeling and subsequent cathodoluminescence (CL) imaging in fine-scale growth studies of echinoderms.

**Results:**

Three species of sea urchins (*Paracentrotus lividus*, *Echinometra* sp. and *Prionocidaris baculosa*) were incubated for different periods of time in seawater enriched in different Mn^2+^ concentrations (1 mg/L; 3 mg/L; 61.6 mg/L). Labeling with low Mn^2+^ concentrations (at 1 mg/L and 3 mg/L) had no effect on behavior, growth and survival of sea urchins in contrast to the high Mn^2+^ dosage (at 61.6 mg/L) that resulted in lack of skeleton growth. Under CL, manganese produced clearly visible luminescent growth fronts in these specimens (observed in sectioned skeletal parts), which allowed for a determination of the average extension rates and provided direct insights into the morphogenesis of different types of ossicles. The three species tend to follow the same patterns of growth. Spine growth starts with the formation of microspines which are simultaneously becoming reinforced by addition of thickening layers. Spine septa develop via deposition of porous stereom that is rapidly (within less than 2 days) filled by secondary calcite. Development of the inner cortex in cidaroids begins with the formation of microspines which grow at ~3.5 μm/day. Later on, deposition of the outer polycrystalline cortex with spinules and protuberances proceeds at ~12 μm/day. The growth of tooth can be rapid (up to ~1.8 mm/day) and starts with the formation of primary plates (pp) in plumula. Later on, during the further growth of pp in aboral and lateral directions, secondary extensions develop inside (in chronological order: lamellae, needles, secondary plate, prisms and carinar processes), which are increasingly being solidified towards the incisal end. Interradial growth in the ambital interambulacral test plates exceeds meridional growth and inner thickening.

**Conclusions:**

Mn^2+^ labeling coupled with CL imaging is a promising, low-cost and easily applicable method to study growth dynamics of echinoderms at the micro-length scale. The method allowed us to evaluate and refine models of echinoid skeleton morphogenesis.

## Background

A number of different methodological approaches have been used to study the growth of echinoderms at different length scales. These include indirect examination of natural growth lines and more direct tagging techniques (via plastic tube slipped over the skeleton, tag inserted into a drilled skeleton, passive integrated transponder tags, coded wire tags, fluorochrome chemical markers and stable isotopes) [1–3]. Among these methods, fluorescent dyes, such as tetracycline and calcein, had attracted considerable attention [4–11]. Both of these chemicals bind to calcium ions and are incorporated into the newly formed calcium carbonate skeleton during biomineralization. Under fluorescence microscopy, tetracycline and calcein fluoresce yellow and green, respectively. However, one of the major disadvantages of using fluorochrome markers in fine-scale biomineralization studies of echinoderms is that produced marks are often diffuse, making it very difficult to measure the growth (cf. Fig. 1 in [9]). Notably, using fluorescent dyes, only millimeter-scale growth fractions can be detected. Furthermore, such chemical markers may stress echinoderms and perturb biomineralization [10]. Recently, Gorzelak et al. [2, 3] introduced a new method of labeling the growing echinoderm skeletons with stable isotope ^26^Mg and NanoSIMS imaging at sub-micrometer spatial resolution. However, although NanoSIMS provides a level of sensitivity unmatched by any other techniques (spatial resolution up to about ~50 nm), it is not a widely accessible analytical tool and the cost of stable isotope is high. Thus, a low-cost, highly efficient and more easily applicable method is still desired for biomineralization studies of echinoderms.

**Fig. 1:**
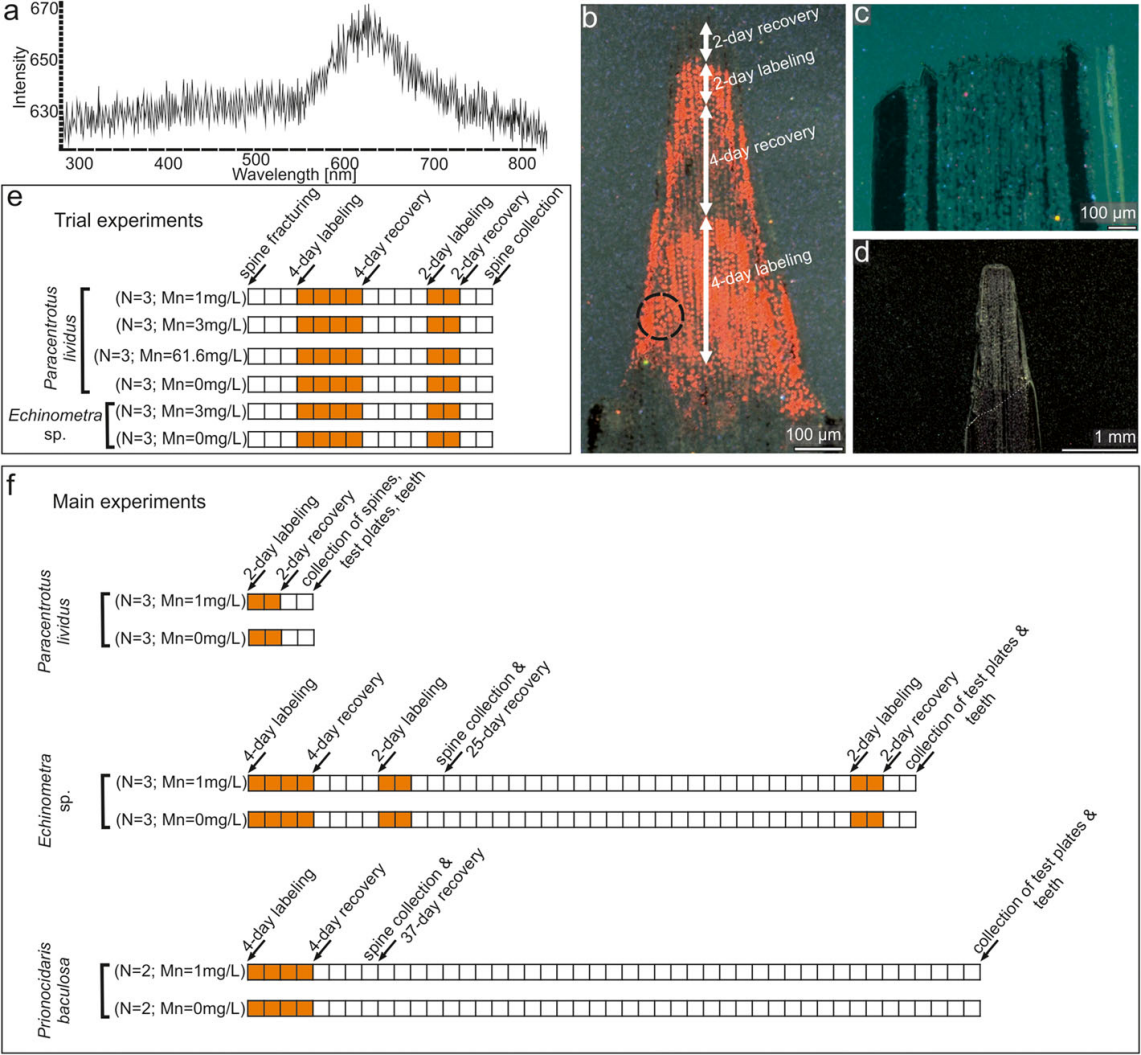
Research methods and design of experiments. **a** example of CL-activated UV–VIS spectrum of a fragment of luminescent spine (marked with a black circle in (**b**) of *Echinometra* sp. labeled with Mn^2+^ at 3 mg/L showing Mn^2+^ emission maximum at 632 nm (Mn^2+^ activation in Ca^2+^ and Mg^2+^ positions in calcite), **b** lateral view of spine showing no growth differences during and between labeling events (orange-red skeletal regions indicate enhanced Mn^2+^ concentrations due to twice Mn^2+^ labeling; dark ragions indicate growth in normal (without Mn^2+^) seawater), **c** lateral view of spine of *Paracentrotus lividus* labeled with Mn^2+^ at 61.4 mg/L without any signs of regeneration, **d**, lateral view of spine of control specimen of *Paracentrotus lividus* without any signs of luminescence, cutting fracture is delineated with dotted line, experimental design of trial (**e**) and main (**f**) experiments; each box represents a 24 h period

Cathodoluminescence is the emission of a photon of characteristic wavelengths during excitation by an incident electron bombardment. CL-luminescence properties of calcite are usually attributed to different proportions of incorporated Mn^2+^ replacing Ca^2+^ in the crystal lattice. Mn^2+^ is considered the most important activator element whereas Fe^2+^ is the most important quencher element in carbonates [12]. The activation of Mn^2+^ in calcite can easily be recognized by a broad emission band in the yellow-orange range (wavelength range: 605–620 nm). However, in the case of echinoderm Mg-calcite, Mn^2+^ typically substitutes two different lattice positions (i.e., Ca^2+^ position in Mg-free calcite „main-structure” and Mg^2+^ position in magnesite-like „sub-structure”; [13, 14]) leading to the shift of Mn^2+^ peak to higher wavelenghts (orange-red emission band centered at ~632 nm, see Fig. 1a, b).

Concentration of mangenese in seawater is typically very low (0.1–0.2 μg/L; see [15, 16]). Therefore, recent biogenic carbonates, although not without exceptions, are typically non-luminescent or very weakly luminescent [17–26]. Owing to this fact, labeling with enhanced Mn^2+^ concentration in seawater proved to be a useful method in studying growth dynamics of some invertebrates, in particular some species of molluscs [19–21]. With respect to echinoderms, it has been argued that most of them are non-luminescent or reveal light blue zones of intrinsic luminescence [12, 18, 23, 27] although it has been shown that test plates of some field collected echinoids from offshore environments may also display orange luminescence zoning [28].

In this paper, we test the feasibility of Mn-labeling method for studying growth dynamics of echinoderms for the first time. Our results provide new insights into the morphogenesis of various types of echinoderm ossicles.

## Methods

Mn-labeling experiments were performed in the Laboratory of Marine Biology in Bruxelles (Université Libre de Bruxelles, Belgium) using three species of sea urchins (*Paracentrotus lividus*, *Echinometra* sp. and *Prionocidaris baculosa*). Specimens of *Paracentrotus lividus*, 2.5–3.5 cm in ambital diameter, were obtained from the aquaculture facility in Luc-sur-Mer (English Channel, Normandy, France). Before the beginning of experiments the specimens were transported to the Laboratory of Marine Biology in Bruxelles and maintained for about two weeks in an aerated, closed circuit aquarium, containing ~1000 l of natural seawater under controlled and constant temperature (~16 °C), salinity (~33.5 psu), and pH conditions (~8.0), similar to the conditions in the aquaculture facility. Commercially obtained specimens of *Echinometra* sp. (3–3.5 cm in ambital diameter) and *Prionocidaris baculosa* (~1.5 cm in ambital diameter) collected from the coastal regions of Indonesia (presice locality unknown) and Cebu in the Philippines, respectively, were maintained for at least 2 weeks in two aerated aquariums, each containing about 100 l of natural seawater under constant temperature (~25.5 °C), salinity (~35 psu), and pH conditions (~8).

To test feasibility of the Mn-labeling method for studying growth dynamics of sea urchins, trial experiments on regenerating spines of *Paracentrotus lividus* and *Echinometra* sp. were conducted first. These initial experiments involved the following steps: (1) spine fracturing; (2) 3 days (72 h) recovery; (3) 4 days (96 h) Mn^2+^ labeling experiment; (4) 4 days (96 h) recovery; (5) 2 days (48 h) Mn^2+^ labeling experiment; (4) 2 days (48 h) recovery after which the experiment ended and the spines were removed from the tests. These steps are explained in details below.

Twelve specimens of *Paracentrotus lividus* and 6 specimens of *Echinometra* sp. were prepared 3 days before the beginning of the labeling experiment by cutting distal tips of three long primary spines. Trial Mn-labeling experiments started 3 days after cutting the spine tips when the process of their regeneration initiated. Twelve specimens of *Paracentrotus lividus* were incubated separately for 4 days in small beakers, each containing 1 l of natural seawater mixed with different concentrations of dissolved MnCl_2_, 4H_2_O (Sigma-Aldrich) resulting in 4 different nominal Mn^2+^ concentrations (control seawater without added Mn^2+^, 1 mg/L, 3 mg/L, 61.6 mg/L), with 3 individuals per concentration. Low concentrations (1 mg/L and 3 mg/L) were used because they seemed to be sufficiently effective to enable mark detection under CL [17]. Importantly, it was previously demonstrated that higher Mn concentrations may induce mortality, stress, developmental defects or even growth inhibition in echinoderms [29–32]. For comparison purpose, we also used a very high manganese concentration (61.6 mg/L) which in previous reports was shown to prevent skeleton growth in echinoid embryos ([29–31]; see also Fig. 1c).

The beakers were covered by hard plastic covers and equipped with an air bubbling system. The specimens of *Echinometra* sp. were only incubated at Mn^2+^ = 3 mg/L and control seawater without added Mn^2+^(3 individuals per concentration). After the first incubation period, all sea urchins were transferred to new beakers containing 1 l of natural seawater without Mn^2+^. After 4 days of recovery, a second 2-day labeling procedure was repeated using the same Mn^2+^ concentrations. Finally, the specimens were again returned to normal conditions for 2 days, after which the experiment was terminated and selected spines with regenerated tips were removed from the urchins (Fig. 1e).

Dissected spines were first soaked in NaOH 1 M for 2 h, and then in 2.5% NaClO for 15 min in order to remove soft tissues. They were then rinsed in MilliQ water for 90 s and dried at 50 °C for ~48 h. Variously oriented thin sections were prepared by polishing the spines down to about ~25 μm. Thin sections were finally coated with carbon and examined with a cathodoluminescence (CL) microscope at the Institute of Paleobiology of the Polish Academy of Sciences in Warsaw. This microscope, linked to a Kappa video camera for recording images, is equipped with a hot cathode and is integrated with UV-VIS spectograph. An electron energy of 14 keV and, depending on a sample, beam currents between 0.7–0.15 mA were used for both CL microscopy and spectroscopy. The exposure time for recording images was about 3 s. Integration time for CL-emission spectra of luminescent ossicles was 100 s.

During the trial experiments all sea urchins labeled with low concentration of manganese (1 mg/L and 3 mg/L) were actively relocating within beakers, did not loose any spine and their podia were actively moving, which suggested that the animals were probably not significantly stressed by increased level of Mn^2+^ as supported by very similar growth rates during and between labeling events (Fig. 1b, see Results section). However, sea urchins subjected to high Mn^2+^ concentration (61.6 mg/L) were clearly stressed, in that growth was ceased (Fig. 1c), some spines were lost and no moving with podia was observed.

**Fig. 2:**
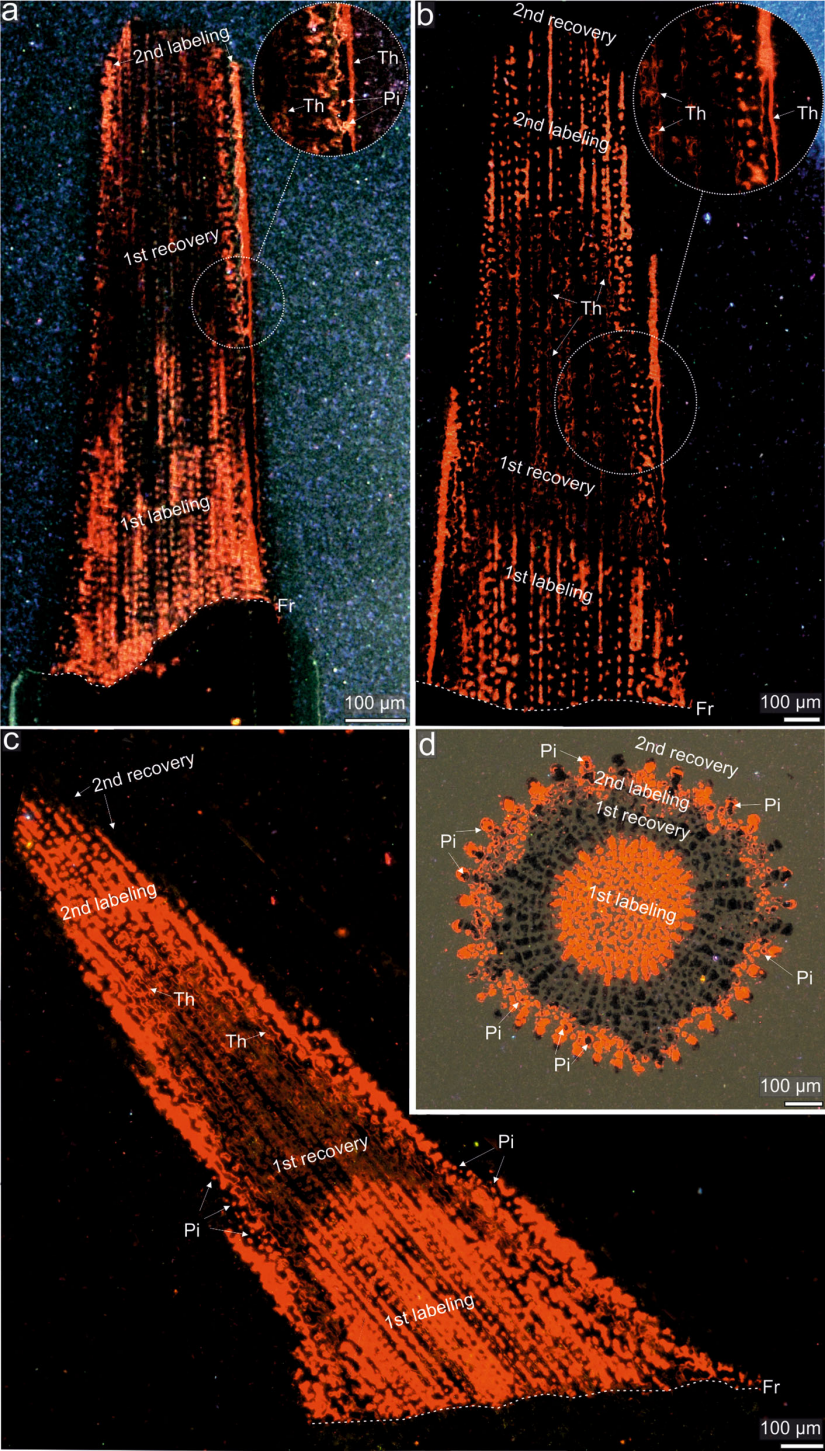
CL images of polished sea urchin spines labeled during trial marking experiments. **a** lateral view with enlarged microregion of a spine showing regenerated tip of *Paracentrotus lividus* labeled with Mn^2+^ at 1 mg/L, **b** lateral view with enlarged microregion of a spine showing regenerated tip of *Paracentrotus lividus* labeled with Mn^2+^ at 3 mg/L, **c** lateral view of spine showing a regenerated tip of *Echinometra* sp. labeled with Mn^2+^ at 3 mg/L, **d** cross-section of a regenerated tip of spine of *Echinometra* sp. labeled with Mn^2+^ at 3 mg/L. Abbreviations: Th - thickening increments, Pi - pore infilling deposit Fr - cutting fracture (delineated with dotted line). Orange-red skeletal regions indicate enhanced Mn^2+^ concentrations due to twice Mn^2+^ labeling; dark regions indicate growth in normal (without Mn^2+^) seawater

Having shown that low concentration of Mn^2+^ appears to be optimal for mark detection in that the two labeling events in the pilot samples were clearly visible under CL imaging (Fig. 2), we performed additional labeling experiments to further explore growth dynamics and morphogenesis of different ossicle types in three different sea urchin species. For this purpose, we used 9 new specimens of *Paracentrotus lividus*, 6 specimens of *Echinometra* sp.*,* which were previously used in the above trial experiments, and 4 new specimens of *Prionocidaris baculosa* (Fig. 1f)*.* The specimens were incubated for either 2 (*Paracentrotus lividus* and *Echinometra* sp.) or 4 (*Prionocidaris baculosa*) days in seawater with the low Mn^2+^ concentrations (1 mg/L). After this immersion period, the specimens of *Paracentrotus lividus* and *Echinometra* sp. were transferred for 2 days to beakers containing normal seawater without Mn^2+^. They were then sacrificed and 3 types of ossicles (non-regenerative spine, tooth and interambulacral ambital test plate from each individual) were prepared for CL analyses in a manner similar to that described above. After 4-days labeling of the specimens of *Prionocidaris baculosa*, they returned to normal conditions for 4 days after which a few primary spines were removed from the urchins and prepared for CL analyses. These individuals were sacrificed after 41 days from the date of the end of Mn-labeling after which interambularal test plates and teeth were also collected for additional CL analyses.

During all labeling experiments, the specimens of *Paracentrotus lividus* were kept at 17 °C, whereas the specimens of *Echinometra* sp. and *Prionocidaris baculosa* were maintained at 25 °C (Table 1). All specimens were kept under 12 h–light/12 h–dark photoperiod. Three parameters (temperature, salinity, and electromotive force- emf) were monitored three times per day using respectively a WTW Multi 340i multimeter equipped with a conductivity cell and integrated temperature sensor and a Metrohm pH-meter (826 pH mobile) equipped with a microelectrode (reference 6.0224.100, Metrohm), and calibrated with CertiPUR® buffer solutions pH 4.00 and 7.00 (Merck, Darmstadt, Germany). All emf measurements were converted to pH in total scale according to DelValls and Dickson’s [33] method with TRIS/AMP buffers (kindly provided by the Biogeochemistry and Earth System Modeling Laboratory of the Université Libre de Bruxelles, Belgium). Differences between these parameters were assessed using one-way ANOVA with Tukey’s multiple comparison tests. These parameters were very comparable and constant throughout all experiments (Table 1). Seawater in each beaker was replaced every day in all the above experiments. Alkalinity values (ranging from 2.4 to 2.6 mmol/kg) were determined by a potentiometric titration with HCl 0.1 M with 0.7 M NaCl using a Titrino 718 STAT Metrohm (Switzerland), and were calculated using the Gran function [34].

**Table 1:** Conditions (mean values) in beakers during trial and main experiments

Trial experiment	Species	Temperature	SD	Salinity	SD	pH_T_	SD
	*Paracentrotus lividus*	15,88	1,07	33,45 ^a^	0,13	7,95	0,09
Controls	*Paracentrotus lividus*	15,83	1,12	33,51	0,13	7,96	0,09
	*Paracentrotus lividus*	15,79	1,1	33,53	0,16	7,95	0,11
	*Paracentrotus lividus*	15,77	1,11	33,52	0,11	7,94	0,9
Mn 1 mg/L	*Paracentrotus lividus*	15,73	1,12	33,51	0,12	7,95	0,1
	*Paracentrotus lividus*	15,68	1,13	33,56	0,13	7,95	0,11
	*Paracentrotus lividus*	15,78	1,12	33,56	0,1	7,96	0,11
Mn 3 mg/L	*Paracentrotus lividus*	15,67	1,16	33,59	0,14	7,97	0,12
	Paracentrotus *lividus*	15,81	1,14	33,56	0,12	7,96	0,12
	*Paracentrotus lividus*	15,84	1,15	33,58	0,1	7,97	0,11
Mn 61.6 mg/L	*Paracentrotus lividus*	15,86	1,15	33,59	0,1	7,95	0,12
	*Paracentrotus lividus*	15,84	1,15	33,59	0,1	7,96	0,11
Main experiment							
	*Paracentrotus lividus*	17,75	0,39	33,48	0,21	8,11	0,06
Controls	*Paracentrotus lividus*	17,69	0,43	33,53	0,24	8,1	0,06
	*Paracentrotus lividus*	17,73	0,35	33,54	0,26	8,11	0,06
	*Paracentrotus lividus*	17,58	0,41	33,52	0,2	8,11	0,06
Mn 1 mg/L	*Paracentrotus lividus*	17,67	0,38	33,51	0,16	8,1	0,07
	*Paracentrotus lividus*	17,57	0,42	33,58	0,36	8,11	0,07
	*Paracentrotus lividus*	17,56	0,34	33,62	0,28	8,09	0,08
Mn 3 mg/L	*Paracentrotus lividus*	17,43	0,46	33,63	0,32	8,11	0,06
	*Paracentrotus lividus*	17,45	0,42	33,63	0,3	8,11	0,06
	*Echinometra* sp.	25,05	0,54	34,46	0,17	8,1	0,06
Controls	*Echinometra* sp.	25,04	0,52	34,48	0,2	8,1	0,05
	*Echinometra* sp*.*	25,06	0,51	34,43	0,2	8,11	0,06
	*Echinometra* sp.	25,06	0,51	34,43	0,19	8,11	0,06
Mn 1 mg/L	*Echinometra* sp.	25,07	0,52	34,47	0,24	8,1	0,05
	*Echinometra* sp.	25,07	0,52	34,45	0,23	8,09	0,05
Controls	*Prionocidaris baculosa*	25,16	0,37	35,29	0,22	7,96	0,05
	*Prionocidaris baculosa*	25,1	0,37	35,29	0,22	7,96	0,05
Mn 1 mg/L	*Prionocidaris baculosa*	25,16	0,36	35,3	0,21	7,95	0,06
	*Prionocidaris baculosa*	25,09	0,36	35,3	0,21	7,95	0,06

SD = standard deviations (*n* = 36). Conditions within individual beakers for a given experiment were not significantly different from each other, except the salinity in a beaker no 1. (marked with ^a^) which is significantly different from each other

Before the beginning of all labeling experiments, the sea urchins were fed ad libitum with Sea Urchin Diets (Zeigler Bros) but during trial and further growth experiments all specimens were fed by only one Zeigler pellet every two days.

Microstructural details of selected samples, etched in 0.1% formic acid solution, dried and coated with carbon [35], were observed in a Phillips XL30 scanning electron microscope (SEM) at the Institute of Paleobiology, Polish Academy of Sciences in Warsaw.

Although our research was focused on qualitative aspects of morphogenesis of various ossicle types, some quantitative data, e.g., the average extension rates during and between labelings (expressed in μm/day) were also collected. These data were analyzed with the Wilcoxon two-sample paired signed rank tests in PAST [36] due to not normally distributed data. We set the significance level to α = 0.05. Thin sections are housed at the Institute of Paleobiology, Polish Academy of Sciences, Warsaw (ZPALV.42Mn-CL).

## Results

### Trial experiments

The lengths of regenerated tips in *Paracentrotus lividus* and *Echinometra* sp. measured from SEM microphotographs were highly variable between spines of the same individual, between individuals within the same treatments, and between individuals from different treatments (Tables 2, 3). The average longitudinal extension rates of regenerating spines in controls and specimens labeled with low Mn^2+^ concentration (1 and/or 3 mg/L) were rather high (Tables 2, 3). On the other hand, no regeneration was observed in all specimens labeled in seawater with the highest Mn^2+^ concentration (61.6 mg/L).

**Table 2:** Lengths of spine tips of *Paracentrotus lividus* regenerated during trial experiments (3 spines per individual/ 3 individuals per treatment) and calculated average longitudinal extension rates (ALER) per treatment

	Mn = 1 mg/L	Mn = 3 mg/L	Mn = 61.4 mg/L	Mn = 0 mg/L
Spine1	2581 mm	1819 mm	0 mm	2491 mm
Spine2	2435 mm	2,52 mm	0 mm	2774 mm
Spine3	2087 mm	2687 mm	0 mm	2756 mm
Spine1	1,65 mm	1,45 mm	0 mm	2457 mm
Spine2	1,69 mm	2,67 mm	0 mm	2867 mm
Spine3	1,83 mm	1,61 mm	0 mm	2894 mm
Spine1	1118 mm	0,922 mm	0 mm	1911 mm
Spine2	3414 mm	3462 mm	0 mm	2313 mm
Spine3	2923 mm	1386 mm	0 mm	2648 mm
ALER	183 μm/day	172 μm/day	0 μm/day	214 μm/day

**Table 3:** Lengths of the spine tips of *Echinometra* sp. regenerated during trial experiments (3 spines per individual/3 individuals per treatment) and calculated average longitudinal extension rates (ALER) per treatment

	Mn = 3 mg/L	Mn = 0 mg/L
Spine1	0,481 mm	4742 mm
Spine2	0,053 mm	1806 mm
Spine3	1929 mm	1835 mm
Spine1	1367 mm	0,083 mm
Spine2	1894 mm	0,12 mm
Spine3	1948 mm	0,05 mm
Spine1	2521 mm	2387 mm
Spine2	4656 mm	2305 mm
Spine3	5,32 mm	2262 mm
ALER	187 μm/day	144 μm/day

**Fig. 3:**
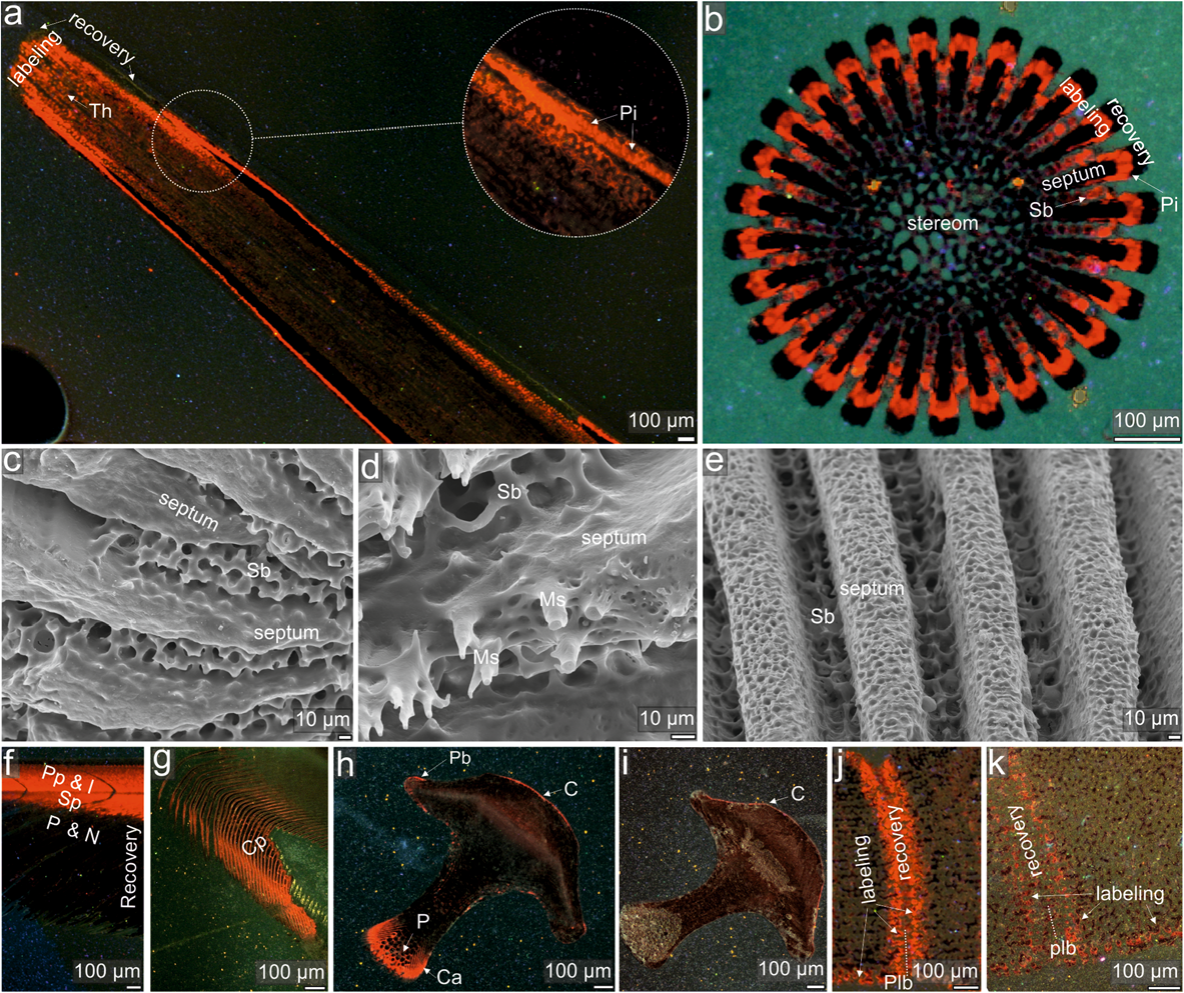
CL and SEM images of *Paracentrotus lividus* labeled with Mn^2+^ at 1 mg/L during the second labeling experiment. **a** lateral view of a polished spine with enlarged microregion showing septa formation, **b** cross-section of a polished spine, **c**-**e** micromorphology of septa revealed after formic acid etching, **f**, **g** cross-sections showing lateral view of the polished tooth, **h**, **i** transverse cross-sections through the polished tooth (at the level of proximal shaft (∼6 mm from the aboral end and ∼8 mm from the adoral tip) and near the incisal end (∼11 mm from the aboral end and ∼3 mm from the adoral tip), respectively), **j**, **k** cross-sections near the polished ambital plates showing a contact between two test plates (adapical and interadial sutures, respectively). Abbreviations: Th - thickening increments, Pi - pore infilling deposit, Ms - microspines, Sb - stereom bridges, C - outer crust, Pb - pillar bridges, P - pisms, Cp - carinar process plates, Sp - secondary plates, Plb - plate boundary, L - lamellae, N - needles. Orange-red skeletal regions indicate enchanced Mn^2+^ concentrations due to Mn^2+^ labeling; dark regions indicate growth in normal (without Mn^2+^) seawater

Figure 2 summarizes results of CL analyses of trial experiments showing examples of spines sectioned either along (Fig. 2 a-c) or perpendicular (Fig. 2d) to the long axis. Under CL, all regenerated spines of both species labeled with low Mn^2+^ concentrations (1 mg/L; Fig. 2a or 3 mg/L; Fig. 2 b-d) reveal two bright orange-red luminescent stereom increments that are separated by one area with dark stereom trabeculae which only occasionally display luminescent outermost thickening trabecular layers. In the peripheral part of the spine, where septa are being formed, the skeleton shows irregular patchy luminescence with some brighter or darker spots. This is especially well visible in transverse sections (Fig. 2d).

The emission spectra of the luminescent regions peaked at ~632 nm (Fig. 1a), consistent with manganese-activated luminescence in echinoderm Mg-calcite [13]. By contrast, the spines with regenerated tips from control specimens are entirely non-luminescent (Fig. 1d). Thus, the two observed luminescent increments alternated with two non-luminescent growth fronts correspond to the two successive Mn-labeling events (4-day and 2-day) and two recovery periods (4-day and 2-day), respectively. As stressed above, no growth increments above the cutting fracture were observed in the spines of specimens incubated in seawater with the highest Mn^2+^ concentration (61.6 mg/L) (Fig. 1c). Under CL, these spines are entirely non-luminescent.

### Main experiments

**Fig. 4:**
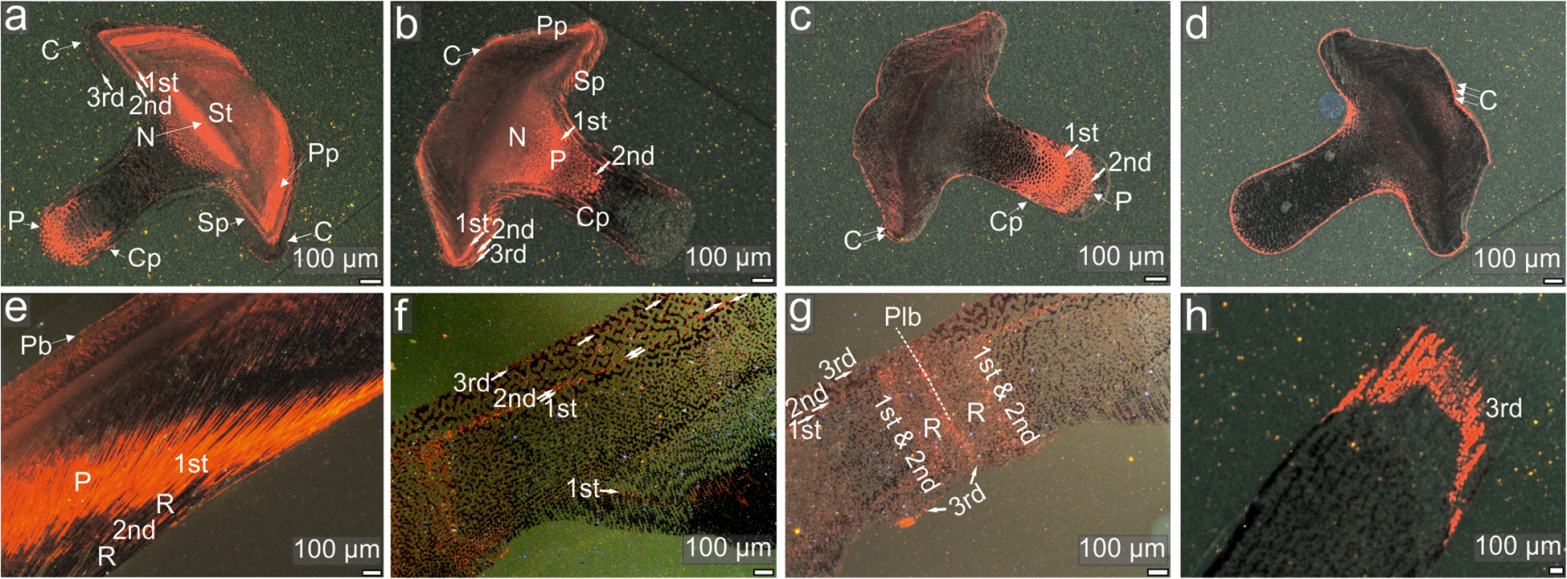
CL images of polished ossicles of *Echinometra* sp. labeled with Mn^2+^ at 1 mg/L during the second labeling experiments. **a**-**d** transverse cross-sections through a tooth (from the level near shaft/plumula boundary (∼8 mm from the adoral tip) towards the incisal end (∼6, 4 and 2 mm from the adoral tip, respectively)), **e** lateral view of tooth near the proximal part of shaft, **f** cross-section of an ambital interambulacral plate in lateral view, **g** cross-section near an ambitus showing a contact (adapical suture) between two test plates, **h** lateral view of a growing spine. Abbreviations: 1st, 2nd, 3rd - successive labeling events (indicated by solid arrows); R - interlabeling recovery, C - outer crust, Pb - pillar bridges, P - pisms, Cp - carinar process plates, Sp - secondary plates, St - stone part, Pp - primary plates, Plb - plate boundary, L - lamellae, N - needles. Orange-red skeletal regions indicate enhanced Mn^2+^ concentrations due to Mn^2+^ labeling; dark regions indicate growth in normal (without Mn^2+^) seawater

**Fig. 5:**
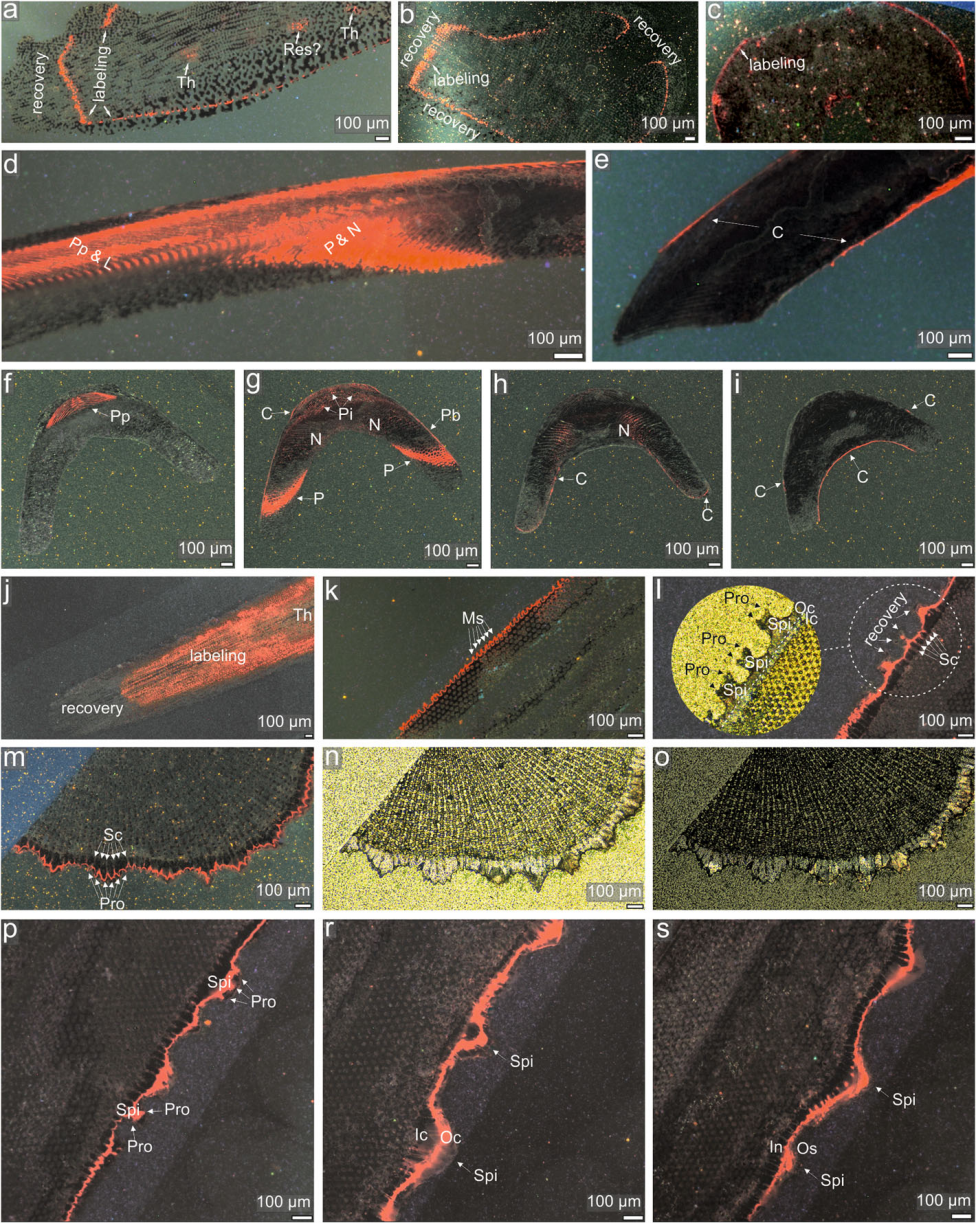
CL and optical images of polished ossicles of *Prionocidaris baculosa* labeled with Mn^2+^ at 1 mg/L during the second marking experiments. **a** cross-section of an ambitus interambulacral plate in lateral view, **b** cross-section of a young interambulacral plate near the apical disc in meridional view, **c** cross-section of an ambitus interambulacral plates with enlarged central tubercle in lateral view, **d** lateral view of tooth near the proximal part of the shaft, **e** lateral view of tooth near the incisal end, **f**-**i** transverse cross-sections through a tooth (from the level near shaft/plumula boundary (∼7 mm from the adoral tip) towards the incisal end (∼5, 3 and 1 mm from the adoral tip, respectively)), **j** lateral view of a distal end of growing spine, **k** lateral view of a growing spines showing an initial phase of cortex formation at the middle height of the spine, **l** lateral view (at the middle height of the spine) of a growing spines showing later phase of cortex formation with enlargement of selected microregion, **m**-**o** cross-sections of a growing spine showing later phase of cortex formation (at the middle height of spine) under CL, optical and polarizing microscope views, respectively, **p**-**s** lateral views (at the middle height of spine) of a growing spine showing late phase of cortex formation. Abbreviations: Th - thickening increments, Res? - possible deposition of calcite on previously resorbed stereom, Ms - microspines, C - outer crust, Pb - pillar bridges, P - prisms, Pp - primary plates, L - lamellae, N - needles, Spi - spinule, Pro - protuberances, Sc - stromatic channels, Ic - inner cortex, Oc - outer cortex. Orange-red skeletal regions indicate enchanced Mn^2+^ concentrations due to Mn^2+^ labeling; dark regions indicate growth in normal (without Mn^2+^) seawater

Figures 3, 4 and 5 show distribution of Mn-induced luminescent increments in various ossicle types of three sea urchin species (*Paracentrotus lividus*, *Echinometra* sp. and *Prionocidaris baculosa*) that were grown during the further dynamic labeling experiments with low Mn^2+^ concentrations (1 mg/L).

#### Paracentrotus Lividus

A single (2-day) labeling event is clearly distinguishable in all types of examined ossicles. The average longitudinal extension rate for a normal (non-regenerative) spine growth, measured in the central axis, is 106 μm/day (Fig. 3a). In the periphery, continuous luminescent layers corresponding to septa, with patchy appearance in some places, extend down to ca. 13 mm below the level of microspine deposition in the distalmost part of the spine (Fig. 3a). In transverse sections, patchy luminescent parts of the septa and some stereom bridges are visible (Fig. 3b-e).

Distribution and intensity of Mn-induced luminescence in the teeth of *Paracentrotus lividus* is not uniform. In particular, high intensity and rather uniform luminescence is observed near plumula/shaft boundary, where new primary plates with lamellae were being formed (Fig. 3 f). Intense luminescence extends down and encompasses also prisms, needles and carinal processes in the keel (Fig. 3g-i). Luminescence gradually disappears towards the adoral part of the tooth shaft. Herein, luminescence is restricted only to some isolated spaces between needle-prisms, primary, secondary and carinar plates. Most adorally, near the incisal end, an outermost layer corresponding to the abaxial crust is only luminescent (Fig. 3i). The estimated longitudinal extension rate of teeth in this species is ~1.8 mm/day. However, the growth rate of teeth in this and other species examined in this study need to be treated with caution because the least solidified part of the tooth, i.e., plumula, is extremely fragile and tends to be lost during preparation of thin sections. Furthermore, non-uniform luminescence due to simultaneous thickening process in adoral direction makes it difficult to measure the growth.

Three luminescent growth fronts are observed in interambulacral ambital plates (Fig. 3j, k). Sharp Mn-label is continuously distributed along the inner plate margin forming a distinct thin layer. Luminescent growth fronts are somewhat more expanded in interradial and meridional directions. Approximated equatorial growth in adambulacral direction (~35 μm/day) exceeds meridional growth (~14 μm/day) and inner plate thickening (~4 μm/day). Luminescence is not observed in external part of the plate, where primary tubercles are present.

#### *Echinometra* sp.

Three labeling events and 3 successive recovery periods (4-day labeling, 4-day recovery, 2-day labeling, 27-day recovery, 2-day labeling and 2-day recovery) are visible in teeth and ambital interambulacral plates under CL (Fig. 4a-g). Distribution and intensity of Mn-induced luminescence in the tooth of *Echinometra* sp. are similar to those observed in *Paracentrotus lividus* (Fig. 4a-e). In the most adoral part of the shaft, however, not only abaxial crustic layer is luminescent but also adaxial outermost layer (Fig. 4d). The longitudinal growth of teeth can be approximated as ~1 mm/day.

Similar to *Paracentrotus lividus* interradial growth of ambital plates in *Echinometra* sp. (~23 μm/day) exceeds meridional growth (~7 μm/day) and inner plate thickening (2 μm/day) (Fig. 4f, g). Likewise, Mn-labels, in a form of continuously distributed layers are present in the inner side of the plate. Luminesent growth fronts are less distinct in interradial and meridional directions, where luminescent newly formed strereom trabeculae merge with the older ones that reveal luminescence only in the outermost thickening stereom layers. Luminescence is also observed externally on the plate surface. However, it commonly encompasses some stereom bars near the plate boundaries, not central tubercles, like mamelons.

The last 2-day labeling event is visible in one of a few examined spines (Fig. 4h). The average longitudinal extension rate for a normal (non-regenerative) spine growth in this species, measured in the central axis, is ~175 μm/day. In contrast to *Paracentrotus lividus* spine, Mn-label does not extend down below deposition of the distalmost stereom microspines.

#### Prionocidaris baculosa

A singe (4-day) labeling event is detectable in all types of ossicles under CL (Fig. 5). Calculated interradial extension rate of ambital plate is ~9 μm/day, whereas meridional growth rate (~3 μm/day) and inner plate thickening (~1 μm/day) are much slower (Fig. 5a). Continuous luminescent growth fronts, encompassing both stereom bars near the plate boundaries (Fig. 5a, b) and central tubercles (Fig. 5c), are also observed on the plate surfaces. Surprisingly, some irregular patches of luminescent stereom or luminescent outer thickening trabecular layers are also present in the central part of the sectioned plate (Fig. 5a). In the case of teeth, the most luminescent areas can be observed near shaft/plumula boundary (Fig. 5d). Herein, a ca. 2 mm in length luminescent growth front, composed of primary plates and lamellae, extends down in adaxial direction to prisms and needles, and to the continuous abaxial and adaxial crustic layers (Fig. 5e-i).

Among 4 examined primary spines, two revealed luminescent part of the cortex at different stage of its development (Fig. 5j-s). The average longitudinal extension rate of the „open stereom” for one of these spine, measured in the central axis, is ~175 μm/day. In this spine, continuous luminescent growth fronts extend from the level of stereom meshwork formation down to the milled ring. Average extension rates for the inner cortical layer and spinule development are ~3.5 μm/day and 12 μm/day, respectively.

#### Comparison of the average extension rates during and between the labeling events

**Fig. 6:**
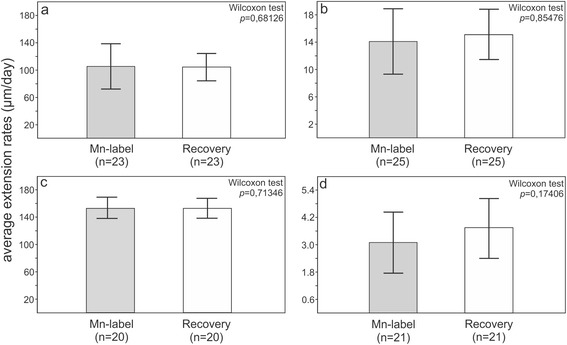
Barcharts showing the average extension rates (±SD) during and between the labeling events for four selected microstructural areas in three examined species. Results of the Wilcoxon tests indicate no statistically significant differences. **a** longitudinal extension rates measured during the first labeling event and subsequent recovery period in *Paracentrotus lividus* (spine illustrated on Fig. 2a), **b** extension rates of test plate in meridional direction during the first labeling event and subsequent recovery period in *Paracentrotus lividus* (plate illustrated on Fig. 3j), **c** longitudinal extension rates of regenerating spine measured during the first labeling event and subsequent recovery period in *Echinometra* sp. (spine illustrated on Fig. 2c), **d** lateral extension rates of inner cortex measured during the first labeling event and subsequent recovery period in *Prionocidaris baculosa* (spine illustrated on Fig. 5m)

In order to test the influence of Mn exposure on growth rates in some ossicles, statistical comparisons between the average extension rates during and between the labeling events in four selected microstructural areas in four ossicles of three examined species were performed (at least 20 measurements per each ossicle in a given direction collected using imageJ). Results clearly show no statistically significant differences (Wilcoxon tests: *p* > 0.05; Fig. 6). Admittedly, however, such estimations of extension rates need to be treated with caution because different orientation of the cut surface may bias interpretations. Furthermore, small portions of the plumula and even potentially distalmost tips of some spines, that were grown during the second recovery period, appeared to be lost during sample preparation, thus at least in the case of spines, comparisons were only made between the skeleton grown during the first labeling event and successive first recovery period.

## Discussion

In the case of labeling mollusc shells most authors recommended to use high-doses of manganese (≥25 mg/L) [19–21, 24–26]. However, care has to be taken because such a high concentration of Mn^2+^ appears to be stressful for sea urchins perturbing their biomineralization [29–31].

Little is known about lower activation limit of Mn^2+^ in calcite. Available data suggest a range 20–1000 ppm [37], much higher than typical skeletal Mn^2+^ content in most echinoderms [38]. As stressed by Richter et al. [12] Mn^2+^ detection limit can be lowered by technical improvements, i.e., by using the so-called hot cathode, instead of the so-called cold cathode. In our labeling experiments we incubated sea urchins with seawater enriched with low concentrations of manganese (1 and/or 3 mg/L) that were subsequently analyzed with the aid of hot cathode. The use of such a low Mn^2+^ concentration and hot cathode in subsequent CL analyses was necessary to avoid stress and growth inhibition [29–31], and to ensure Mn-label detection. Indeed, our data show that the sea urchins labeled at high Mn^2+^ concentrations (61.6 mg/L) were not regenerating their spines. This observation is consistent with previous studies on sea urchin embryo suggesting that that high Mn^2+^ concentrations lead to Ca^2+^ pump dysfunction resulting in a strong depletion of Ca^2+^ in the Golgi regions causing morphological abnormalities (at ≥7.7 mg/L), or inhibition of skeleton growth (at ≥61.6 mg/L) [29–31]. On the other hand, our sea urchins labeled at low Mn^2+^ concentrations (1 and/or 3 mg/L) did not seem to be visibly stressed and went on growing. Notably, the absence of growth-rate differences during and between labeling events suggests that the Mn-labeling did not affect growth rate at least in some of the studied ossicles that were subjected to statistical comparisons (Fig. 6). In order to futher test the effect of Mn-labeling procedure on the sea urchin physiology, future analyses on respiration rates and immunotoxicological studies are planned. However, it seems that larger volumes of seawater, shorter labeling-time scale and lower concentrations of manganese may all minimize potential stress. Notably, our sea urchins labeled at 1 mg/L [Mn^2+^] revealed bright luminescence using a beam current 0.15 mA, an electron energy 14 keV and the exposure time for recording images 3 s. Thus, it seems that labeling sea urchins at much lower concentration can still be detectable by increasing the exposure time and a beam current to the upper limit 0.2 mA. In fact, it has been recently shown that oysters labeled in seawater with low Mn^2+^ concentration (0.017 mg/L), formed a new shell that was barely visible but still detectable by cathodoluminescence [19].

### Implications for morphogenesis of echinoid ossicles

Our study highlights the potential of using a combination of low Mn^2+^ doses in labeling experiments and hot cathode in subsequent CL imaging in biomineralization studies of echinoderms. Admittedly, caution is required in applying this method to all echinoderms because it has been shown that some test plates of Recent echinoids, having increased levels of skeletal Mn, may show CL luminescence [28]. Indeed, although most echinoderms are non-luminescent [18, 27], skeletal concentration of Mn and Fe may differ between species and/or between populations of the same species from different environments [39]. Thus, the use of control specimens is strongly advised to assure validity of CL results.

For the specimens investigated in this study, we demonstrated that this method proved to be successful. This allowed for a much more detailed view of the growth dynamics and morphogenesis of echinoid skeleton at the micro-length scale. In the following we briefly review published data on morphogenesis of echinoderm skeleton and discuss some of the major implications of our results for understanding echinoderm biomineralization.

#### Spine growth

Our data demonstrate that average longitudinal extension rates of regenerating spines in *Paracentrotus lividus* (172 or 183 μm/day depending on a treatment) and *Echinometra* sp. (187 μm /day) are very similar to “normal” non-regenerative spine growth during experiments (106 and 175 μm/day, respectively; cf. Figs. 3a, 4h). Likewise, longitudinal extension rate of primary spine of *Prionocidaris baculosa* (175 μm/day; Fig. 5j) is very comparable. Consistently, previous study also reported that *Paracentrotus lividus* regenerates their spines at similar extension rate (130 μm/day; [2]). Notably, extension rates of spines in other sea urchin species (*Strongylocentrotus purpuratus* (160 μm/day) and *Arbacia punctulata* (260 μm/day)) reported by previous authors [40] are within the same order of magnitude. In three examined species, morphogenesis of stereom meshwork in spine is also basically the same, and follows a growth model of spine based on *Paracentrotus lividus* recently introduced by Gorzelak et al. [2]. Growth of the spine stereom starts with the formation of thin microspines which fuse together at regular intervals by lateral bridges. Simultaneously, thickening of the previously formed stereom trabeculae, in the form of deposition of continuous layers, proceeds on a time scale of about 1 μm/day. Thickening process, involving both longitudinal strereom trabeculae and lateral bridges, may extend down to about 1 mm below deposition of massive microspines. Overall, similar extension rates and stereom morphogenesis in various unrelated echinoid species clearly suggest some common regulatory factors and pathways involved in spine development.

Our data also provided direct insights into the timing and mechanisms of septa development. CL images revealed that septa in *Paracentrotus lividus* and *Echinometra* sp. can be formed via two, but not clearly separated from each other, phases of biomineralization, i.e., deposition of porous stereom that is secondarily filled by calcite as first suggested by [40]. The secondary process of peripheral pore occlusion in radial sectors may proceed rapidly (within less than 2 days) and can be simultaneous with the formation of “open” stereom in the central and peripheral areas, as well as transverse bridges joining the septa. Later on, most proximally the outer part of septa can be thickened by deposition of more or less continuous sheets and layers.

**Fig. 7:**
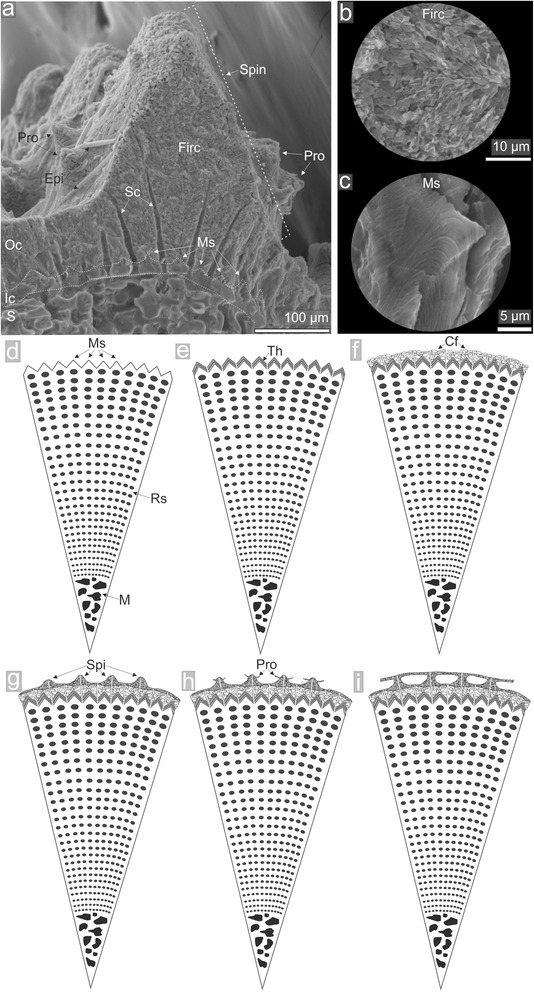
Model of cortex development. **a** SEM image showing basic morphological features of cortex, **b** enlargement of fine irregular polycrystalline structure from the outermost cortex, **c** enlargement of the layered microspine from the inner cortex. **d**-**i** successive phases of cortex formation: microspine development (d), thickening of microspines (e), deposition of more massive stereom with conchoidal fracture on layered microspines between stromatic channels (f), development of spinules (g) formation of protuberances from spinules (h), eventual fusion of neighboring protuberances (i). Abbreviations: Spin - spinule, Pro - proturberances, Epi - epibionts, S - stereom, Ic - inner cortex, Oc - outer cortex, Ms - microspines, Firc - fine irregular crystal structure, Sc - stromatic channels, M - medulla, Rs - rectilinear stereom

In *Prionocidaris baculosa*, external parts of mature spines do not display septa but are covered by the so-called cortex, characteristic for all cidaroids [41, 42]. So far, little was known about morphogenesis and growth dynamics of this structure. Dery et al. [43] based on SEM comparisons of different spines removed from cidaroid tests hypothesized the following steps in cortex development: (i) microspines formation, (ii) successive thickening of microspines by addition of lamellar layers, (iii) spinule development by further thickening of microspines in selected regions, (iv) formation of lateral protuberances eventually fusing between adjacent spinules. Our data proved that cidaroid cortex indeed starts with formation of microspines which are gradually becoming thickened externally (Fig. 7). Later on, the outer cortex, revealing conchoidal fracture in the bottom and the overlaying fine irregular crystals forming spinules with lateral protuberances, develop. This outermost cortical layer has a polycrystalline nature as revealed by the cross-polarized light (Fig. 5o) [41, 42].

The above data appear to be mostly consistent with the hypothetical developmental model by Dery et al. [43]; nevertheless they also provide first direct insights into spatiotemporal development of cortex. For instance, the cortex develops simultaneously with the formation of new open stereom meshwork in the apical part of the spine. From the level of distalmost stereom meshwork formation, cidaroid cortex, in the form of continuous growth front with regularly spaced pores of stromatic channels, extends down at ~3.5 μm/day along the entire spine surface to the level of the milled ring. Thickening rate can be more pronounced (~12 μm/day) at more or less regular intervals giving rise to spinule development of the so-called outer cortex (Fig. 7) which may extend laterally to form protuberances.

#### Teeth

**Fig. 8:**
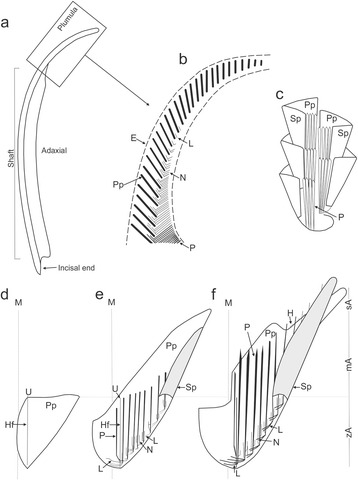
Model of tooth development (compiled and modified after [45–49]). **a** lateral view of a tooth of *Paracentrotus lividus*, **b** enlargement of plumula region, **c** cone-like tooth elements, sticking alternately one within the other, **d**-**f** growth of individual tooth element from initial growth phase (**d**) to late stage (**f**). Abbreviations: E - epidermis, Pp - primary plate, L - lamellae, N - needles, P - prism, Sp - secondary plate, H - pillar bridges, M - median plane, Hf - main fold of pp., U - umbo, zA - central part of pp., mA - middle part of pp., sA - side part of pp.

Morphology and development of echinoid teeth were described extensively in the literature [44–51] but the accurate growth dynamics of individual tooth components are still not well understood. In general, it has been argued that the timing of tooth growth depends on both intrinsic and extrinsic factors. Depending on the species the growth rate may range from 1 to 1.5 mm per week [44, 52]. Furthermore, when food-limited, the growth of echinoid jaws can be much faster [53–55]. In echinoid teeth three zones can be distinguished, i.e., plumula, shaft and incisal end (Fig. 8a). The plumula is the growing zone of the tooth. Herein, triangular parallel arrays of slightly curved primary plates (pp), initially separated from each other, are being formed (Fig. 8b, c). Later on, these plates are thought to grow in aboral and lateral direction to form the “tongue-like” stage from which secondary plates (sp) extend (Fig. 8d-f). Subsequently, sp gradually curve towards the adaxial area and merge to form carinal processes in the keel which is not pronounced in cidaroids. Both pp and sp are stacked within each other resembling ice-cream cones (Fig. 8c). Simultaneously, lamella-needle-prism complexes are being formed from the adhesive points. Lamellae attach to the primary plates near the midline of the flange, whereas needles and prisms protrude axially and proximally at about right angles forming the so-called stone part, and a part of the keel. A tooth zone, where the keel reaches its fullest size, is termed a shaft. Herein, pillar-bridges are mostly present. They are located on the abaxial surfaces to which collagenous tissues attach and connect the tooth with the pyramids (jaws). Towards the distal part, tooth is becoming increasingly compact and solid due to conspicuous progressive thickening of the previously formed plates and infilling of cavity systems by calcite during the second stage of biomineralization. Within the least porous distalmost part of the tooth, in the so-called incisal or oral end, which continuously wears through abrasion during grazing, no mineral deposition is thought to occur.

The above literature data are mostly based on indirect structural observations of teeth removed from different sea urchins. Results presented here thus provide a more direct data on morphogenesis and growth dynamics of echinoid teeth. We demonstrate that the growth of tooth may vary considerably according to the species (~1.8 mm/day in *Paracentrotus lividus*, ~1 mm/day in *Echinometra* sp. and ~0.5 mm/day in *Prionocidaris baculosa*). In general, reported growth rates are higher than those previously reported in the literature (~0.24 mm/day) but this can be attributed to rather food-limited conditions implemented during experiments (one Zeigler pellet per every two days). From the CL images a highly complex growth process becomes apparent. Different tooth elements are beginning to form in a sequential order (primary plates, lamellae, needles, secondary plate, prisms and carinar processes). Later on, however, these elements extend and thicken contemporaneously in different directions and at different rates. CL images revealed that while new plates are being formed in plumula, older structural elements, deeper in the shaft extend and thicken simultaneously. During simultaneous addition of new prisms and carinal processes externally extending the flanges and keel, the older, inner ones at the same height level are simultaneously becoming thickened. The calculated rate of infilling of cavity systems between prisms by calcite in the keel and flanges in available transverse sections intersected at comparable tooth height is within a range of ~1.5–3 μm/day in three examined species. Longitudinal extension rates of individual prisms are highly heterogenous and depends on the level from which they grow. In general, prisms may extend up to about 90 times more than they widen, consistent with theoretical predictions by Robach et al. [56]. Intriguingly, contemporaneous deposition of continuous outermost layers extending down to the distal part of the shaft may also take place. This demonstrates that the odontoblasts can be still active even in the distal part of the tooth, just above the incisal end.

#### Test plate

Growth of echinoid test has been investigated with the aid of radioisotopes and fluorescent markers by many authors [4, 57, 58, 59, 60, 61, 62] whose works inspired other researchers to develop several theoretical growth models of echinoid test [63–73]. According to extensive literature data, new coronar plates, which are added at the apical end of the corona, are subsequently shifted towards the peristome increasing their size in three dimensions. Extension rates of test plates depend on several factors, including the type of plate, their position within the test, and direction of the growth front. In general, circumferential (in interradial direction) growth is known to exceed meridional extension. The maximum interradial growth is usually found in ambital plates whereas maximum meridional growth is found in young plates near the apical disc. The growth of echinoid plates at the macroscale and theoretical framework for understanding various shapes of echinoid tests are well characterized, and thus this was not an ultimate goal of our study. In this study we focused on stereom development in individual test plate (mostly interambulacral ambitals) at the microscale because this aspect is not fully understood.

**Fig. 9:**
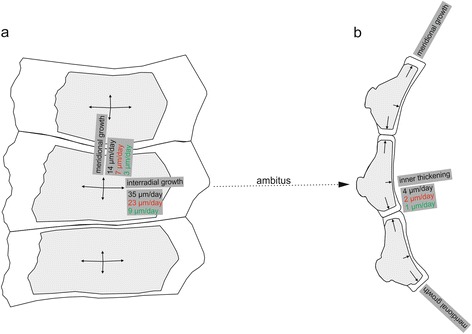
Model of interambulacral ambital plate development (modified after [58]). **a** tangential view, **b** meridional view. Arrows indicate directions of the growth fronts. Mean growth rates per each species in a given direction are highlighted in color (black = *Paracentrotus lividus*, red = *Echinometra* sp., green = *Prionocidaris baculosa*)

We showed that ambital plates thicken very slowly from the inner side. Thickening of test plate proceeds via deposition of continuously distributed layer on previously formed trabeculae. The thickening rate is the slowest in the central part of the plate, and progressively increases towards the plate margins. The growth fronts, in the form of galleried stereom increments, expand more rapidly in interradial and meridional directions (Fig. 9). The three species are characterized by similar growth ratios: interradial/meridional (*Paracentrotus lividus*: 2.4, *Echinometra* sp.: 3.3; *Prionocidaris baculosa*: 3); meridional/thickening (*Paracentrotus lividus*: 3.5, *Echinometra* sp.: 3.5; *Prionocidaris baculosa*: 3); interradial/thickening (*Paracentrotus lividus*: 8.8, *Echinometra* sp.: 11.5; *Prionocidaris baculosa*: 9). In general, the average extension rates of three species examined in this study are comparable to those previously reported in the literature [7, 59]. In some species (*Echinometra* sp.) thickening of the previously formed stereom trabeculae, in the form of deposition of outermost trabecular layers, may also occur. New stereom is rarely formed on external part of the ambital plates where central tubercles, such as mamelons are fully developed. However, in young individuals, such as in the case of *Prionocidaris baculosa* examined in this study, deposition of continuous growth front involving mamelons may also occur on the surface of ambital plates and is obviously more pronounced in the newely formed plates near the apical disc (Fig. 5a, b). It is noteworthy that in *Prionocidaris baculosa* some newly grown isolated stereom increments in small voids or thickening of the previously formed trabeculae can proceed in the central parts of the plate.

## Conclusions

In this paper we introduced a promising method for labeling the growing echinoderm skeleton with manganese coupled with cathodoluminescence imaging to obtain information about biomineralization processes at the micro-length scale. Three sea urchin species were incubated in seawater enriched with Mn^2+^ which is the most important activator element of cathodoluminescence in calcite. Mn^2+^-induced luminescent marks do not diffuse, allowing growth measurements to be made at the microscale resolution. This approach allowed for a refinement of the previous growth models of echinoid spine, tooth and test plate. Spine skeletogenesis begins with the formation of microspines which extend at least 100 times faster than simultaneous thickening process of individual stereom trabeculae. Development of septa proceeds via secondary peripheral pore occlusion in radial sectors. The timescale of formation of septa can be on the order of less than few days. Morphogenesis of cortex in cidaroids starts with the formation of microspines which thicken at ~3.5 μm/day along the entire spine surface to the level of milled ring. Later on, the thickening rate can be more pronounced (~12 μm/day) at more or less regular intervals giving rise to spinule development which may extend laterally to form protuberances. Formation of tooth is a highly complex process and involves formation of new plates in plumula and simultaneous addition of new plates in the shaft extending the keel, and progressive infilling of internal porous structure towards the incisal end. Formation of echinoid ambital plate falls into a readily identifiable pattern: interradial growth exceeds meridional growth and inner thickening process. Mn-labeling coupled with subsequent CL imaging is the first highly efficient method which enables a clear mark detection of the growing echinoid tooth.

## References

[1] Ebert TA, Lawrence JM (2007). Growth and survival of postsettelement sea urchins. Edible sea urchins: biology and ecology.

[2] Gorzelak P, Stolarski J, Dubois P, Kopp C, Meibom A (2011). ^26^Mg labeling of the sea urchin regenerating spine: insights into echinoderm biomineralization process. J Struct Biol.

[3] Gorzelak P, Stolarski J, Dery A, Dubois P, Escrig S, Meibom A (2014). Ultra- and micro-scale growth dynamics of the cidaroid spine of *Phyllacanthus imperialis* revealed by ^26^Mg labeling and NanoSIMS isotopic imaging. J Morphol.

[4] Kobayashi S, Taki J (1969). Calcification in sea urchins I. A tetracycline investigation of growth of the mature test in *Strongylocentrotus intermedius*. Calcif Tissue Res.

[5] Ebert TA (1980). Relative growth of sea urchin jaws: an example of plastic resource allocation. Bull Mar Sci.

[6] Ellers O, Johnson AS (2009). Polyfluorochrome marking slows growth only during the marking month in the green sea urchin *Strongylocentrotus droebachiensis*. Invertebr Biol.

[7] Johnson AS, Salyers JM, Alcorn NJ, Ellers O, Allen DJ (2013). Externally visible fluorochrome marks and allometries of growing sea urchins. Invertebr Biol.

[8] Russell MP (1987). Life history traits and resource allocation in the purple sea urchin *Strongylocentrotus purpuratus* (Stimpson). J Exp Mar Biol Ecol.

[9] Russell MP, Meredith RW (2000). Natural growth lines in echinoid ossicles are not reliable indicators of age: a test using *Strongylocentrotus droebachiensis*. Invertebr Biol.

[10] Russell MP, Urbaniak LM, Heinzeller T, Nebelsick JH (2004). Does calcein affect estimates of growth rates in sea urchins?. Echinoderms. München.

[11] Russell MP, Ebert TA, Petraitis PS (1998). Field estimates of growth and mortality of the green sea urchin, *Strongylocentrotus droebachiensis*. Ophelia.

[12] Richter DK, Goette T, Goetze J, Neuser RD, Neuser RD (2003). Progress inapplication of cathodoluminescence (CL) in sedimentary petrology. Miner Petrol..

[13] Habermann D, Neuser RD, Richter DK, Pagel M, Barbin V, Blanc P, Ohnenstetter D (2000). Quantitative high resolution spectra analysis in sedimentary calcite. Cathodoluminescence in geosciences.

[14] El Ali A, Barbin G, Cervelle B, Ramseyer K, Bouroulec J (1993). Mn^2+^-activated luminescence in dolomite, calcite and magnesite: quantitative determination of manganese and site distribution by EPR and CL spectroscopy. Chem Geol.

[15] Bender ML, Klinkhammer GP, Spencer DW (1977). Manganese in seawater and the marine manganese balance. Deep-Sea Res.

[16] Nordstrom DK, Plummer LN, Wigley TML, Ball JW, Jenne EA, Bassett RL, Crerar DA, Florence TM, Fritz B, Hoffman M, Holdren GR, Lafon GM, Mattigod SV, McDuff RE, Morel F, Reddy MM, Sposito G, Thrailkill J, Jenne EA (1979). Comparison of computerized chemical models for equilibrium calculations in aqueous systems. Chemical modelling in aqueous systems, ACS symposium series 93.

[17] Barbin V, Ramseyer K, Debenay JP, Schein E, Roux M, Decrouez D (1991). Cathodoluminecence of recent biogenic carbonates: an environmental and ontogenic fingerprint. Geol Mag.

[18] Barbin V, Blanc BV, Pagel M, Ohnenstetter D (2000). Cathodoluminescence of carbonates shells: biochemical vs diagenetic process. Cathodoluminescence in geosciences.

[19] Barbin V, Ramseyer K, Elfman M (2008). Biological record of added manganese in seawater: a new efficient tool to mark in vivo growth lines in the oyster species *Crassostrea gigas*. Int J Earth Sci.

[20] Mahé K, Bellamy E, Lartaud F, de Rafélis M (2010). Calcein and manganese experiments for marking the shell of the common cockle (*Cerastoderma edule*): tidal rhythm validation of increments formation. Aquat Living Resour.

[21] Lartaud F, de Rafelis M, Ropert M, Emmanuel L, Geairon P, Renard M (2010). Mn labelling of living oysters: artificial and natural cathodoluminescence analyses as a tool for age and growth rate determination of *C*. *gigas* (Thunberg, 1793) shells. Aquaculture.

[22] Lartaud F, Pareige S, de Rafelis M, Feuillassier L, Bideau M, Peru E, Romans P, Alcala F, Le Bris N (2013). A new approach for assessing cold-water coral growth in situ using fluorescent calcein staining. Aquat Living Resour.

[23] Barbin V (2013). Application of cathodoluminescence microscopy to recent and past biological materials: a decade of progress. Miner Petrol.

[24] Hawkes GP, Day RW, Wallace MW, Nugent KW, Bettiol AA, Jamieson DN, Williams MC (1996). Analyzing the growth and form of mollusc shell layers, in situ, by cathodoluminescence microscopy and Raman spectroscopy. J Shellfish Res.

[25] Langlet D, Alunno-Bruscia M, De Rafelis M, Renard M, Roux M, Schein E, Buestel D (2006). Experimental and natural cathodoluminescence in the shell of *Crassostrea gigas* from Thau lagoon (France): ecological and environmental implications. Mar Ecol Prog Ser.

[26] Auzoux-Bordenave S, Brahmi C, Badou A, de Rafelis M, Huchette S (2015). Shell growth, microstructure and composition over the development cycle of the European abalone *Haliotis tuberculata*. Mar Biol.

[27] Gorzelak P, Krzykawski T, Stolarski J (2016). Diagenesis of echinoderm skeletons: constraints on paleoseawater Mg/Ca reconstructions. Glob Planet Change..

[28] Richter DK, Zinkernagel U (1981). Zur Anwendung der Kathodolumineszenz in der Karbonatpetrographie. Geol Rundsch.

[29] Pinsino A, Matranga V, Trinchella F, Roccheri MC (2010). Sea urchin embryos as an in vivo model for the assessment of manganese toxicity: developmental and stress response effects. Ecotoxicology.

[30] Pinsino A, Roccheri MC, Costa C, Matranga V (2011). Manganese interferes with calcium, perturbs ERK signalling and produces embryos with no skeleton. Toxicol Sci.

[31] Pinsino A, Matranga V, Roccheri MC, Srivastava J (2012). Manganese: a new emerging contaminant in the environment. Environmental Contamination.

[32] Nilsson Sköld H, Baden SP, Looström J, Eriksson SP, Hernroth BE (2015). Motoric impairment following manganese exposure in asteroid echinoderms. Aquat Toxicol.

[33] DelValls TA, Dickson AG (1998). The pH of buffers based on 2-amino-2-hydroxymethyl-1,3-propanediol (‘tris’) in synthetic sea water. Deep-Sea Res Pt I.

[34] Gran G (1952). Determination of the equivalence point in potentiometric titrages-part II. Analyst.

[35] Stolarski J (2003). 3-dimensional micro- and nanostructural characteristics of the scleractinian corals skeleton: a biocalcification proxy. Acta Palaeontol Pol.

[36] Hammer Ø, Harper DAT, Ryan PD (2001). PAST: paleontological statistics software package for education and data analysis. Palaeontol Electron.

[37] Füchtbauer H, Richter DK. Karbonatgesteine. In Füchtbauer H, editor. Sedimente und Sedimentgesteine, Stuttgart: Schweizerbart; 1988. p. 233–434.

[38] Lebrato M, McClintock JB, Amsler MO, Ries JB, Egilsdottir H, Lamare M, Amsler CD, Challener RC, Schram JB, Mah CL, Cuce J, Baker BJ (2013). From the Arctic to the Antarctic: the major, minor, and trace elemental composition of echinoderm skeletons. Ecology.

[39] Bray L, Pancucci-Papadopulou MA, Hall-Spencer JM (2014). Sea urchin response to rising pCO2 shows ocean acidification may fundamentally alter the chemistry of marine skeletons. Mediterr Mar Sci.

[40] Heatfield BM (1971). Growth of the calcareous skeleton during regeneration of spines of the sea urchin *Strongylocentrotus purpuratus* (Stimpson); a light and scanning electron microscope study. J Morphol.

[41] Märkel K, Kubanek F, Willgallis A (1971). Polykristalliner Calcit bei Seeigeln (Echinodermata, Echinoidea). Z Zellforsch.

[42] Märkel K, Roser U (1983). The spine tissues in the echinoid *Eucidaris tribuloides*. Zoomorphology.

[43] Dery A, Guibourt V, Catarino AI, Compère P, Dubois P (2014). Properties, morphogenesis, and effect of acidification on spines of the cidaroid sea urchin *Phyllacanthus imperialis*. Invertebr Biol.

[44] Märkel K (1969). Morphologie der Seeigelzahne. II. Die gekielten Zahne der Echinacea (Echinoder- mata, Echinoidea). Z Morph Tiere.

[45] Märkel K (1970). The tooth skeleton of *Echinometra mathaei* (Blainville) (Echinodermata, Echinoidea). Annot Zool Jap.

[46] Märkel K, Gorny P, Abraham K (1977). Microarchitecture of sea urchin teeth. Fortschr Zool.

[47] Märkel K (1978). On the teeth of the recent cassiduloid *Echinolampas depressa* gray, and on some liassic fossil teeth nearly identical in structure (Echinodermata, Echinoidea). Zoomorphology.

[48] Kniprath E (1974). Ultrastructure and growth of the sea urchin tooth. Calcified Tissue Res.

[49] Ziegler A, Stock SR, Menze BH, Smith AB (2012). Macro- and microstructural diversity of sea urchin teeth revealed by large-scale micro-computed tomography survey. Proc SPIE.

[50] Stock SR, Ignatiev KI, Dahl T, Veis A, De Carlo F (2003). Three-dimensional microarchitecture of the plates (primary, secondary, and carinar process) in the developing tooth of *Lytechinus variegatus* revealed by synchrotron X-ray absorption microtomography (microCT). J Struct Biol.

[51] Stock SR (2014). Sea urchins have teeth? A review of their microstructure, biomineralization, development and mechanical properties. Connect Tissue Res.

[52] Holland ND (1965). An autoradiographic investigation of tooth renewal in the Purple sea urchin (*Strongylocentrotus purpuratus*). J Exp Zool.

[53] Levitan DR (1991). Skeletal changes in the test and jaws of the sea urchin *Diadema antillarum* in response to food limitation. Mar Biol.

[54] Lewis CA, Ebert TA, Boren ME (1990). Allocation of 45calcium to body components of starved and fed purple sea urchins (*Strongylocentrorus purpuratus*). Mar Biol.

[55] Ebert TA, Hernández JC, Clemente S (2014). Annual reversible plasticity of feeding structures: cyclical changes of jaw allometry in a sea urchin. Proc R Soc B.

[56] Robach JS, Stock SR, Veis A (2009). Structure of first- and second-stage mineralized elements in teeth of the sea urchin *Lytechinus variegatus*. J Struct Biol.

[57] Pearse JS, Pearse VB (1975). Growth zones in the echinoid skeleton. Am Zool.

[58] Märkel K (1975). Wachstum des Coronarskeletes von *Paracentrotus lividus* Lmk. (Echinodemmata, Echinoidea). Zoomorphology.

[59] Märkel K (1981). Experimental morphology of coronar growth in regular echinoids. Zoomorphology.

[60] Dafni J (1986). A biomechanical model for the morphogenesis of regular echinoid tests. Paleobiol..

[61] Ebert TA (1988). Allometry, design and constraint of body components and of shape in sea urchins. J Nat Hist.

[62] Gage JD (1991). Skeletal growth zones as age-markers in the sea urchin *Psammechinus miliaris*. Mar Biol.

[63] Thompson DAW (1917). On growth and form.

[64] Moss ML, Meehan M (1968). Growth of the echinoid test. Acta Anat.

[65] Raup DM (1968). Theoretical morphology of echinoid growth. J Paleontol.

[66] Seilacher A (1979). Constructional morphology of sand dollars. Paleobiol.

[67] Telford M (1985). Domes, arches and urchins: the skeletal architechture of echinoids (Echinodermata). Zoomorphology.

[68] Telford M (1994). Structural models and graphical simulation of echinoids.

[69] Baron CJ. What functional morphology cannot explain: a model of sea urchin growth and a discussion of the role of morphogenetic explanations in evolutionary biology. In: Dudley EC, editor. The unity of evolutionary biology. Proceedings of the Fourth International Congress of Systematic and Evolutionary Biology. Dioscorides. Portland; 1990. p. 471–488.

[70] Ellers O. A mechanical model of growth in regular sea urchins: predictions of shape and a developmental morphospace. Proc R Soc Lond B Biol Sci. London. 1993;254:123–9.

[71] Zachos LG. An equilibrium theory of echinoid plate geometry. GSA Abstracts with Programs 2007;39:501.

[72] Zachos LG (2009). A new computational growth model for sea urchin skeletons. J Theor Biol.

[73] Abou Chakra M, Stone JR (2011). Holotestoid: a computational model for testing hypotheses about echinoid skeleton form and growth. J Theor Biol.

