# Correction to: Sea urchin growth dynamics at microstructural length scale revealed by Mn-labeling and cathodoluminescence imaging

**DOI:** 10.1186/s12983-018-0253-1

**Published:** 2018-04-12

**Authors:** Przemysław Gorzelak, Aurélie Dery, Philippe Dubois, Jarosław Stolarski

**Affiliations:** 1 Institute of Paleobiology, Polish Academy of Sciences, Twarda 51/55, 00-818 Warsaw, Poland; 2 Laboratoire de Biologie marine, Faculté des Sciences, Université Libre de Bruxelles, CP 160/15, av., F.D.Roosevelt, 50, B-1050 Bruxelles, Belgium

## Correction

Upon publication of this article [1] it was noticed errors were introduced during the production process which resulted in the omission of decimal points from Tables 2 and 3. In addition Table 1 incorrectly used commas to indicate decimal points. These errors do not change the scientific conclusions of the article in any way. The publisher apologizes for these errors. The correct versions of Tables 1, 2 and 3 appear below and have been updated in the original article.

**Table 1:** Conditions (mean values) in beakers during trial and main experiments

Trial experiment	Species	Temperature	SD	Salinity	SD	pH_T_	SD
	*Paracentrotus lividus*	15.88	1.07	33.45*	0.13	7.95	0.09
Controls	*Paracentrotus lividus*	15.83	1.12	33.51	0.13	7.96	0.09
	*Paracentrotus lividus*	15.79	1.1	33.53	0.16	7.95	0.11
	*Paracentrotus lividus*	15.77	1.11	33.52	0.11	7.94	0.9
Mn 1mg/L	*Paracentrotus lividus*	15.73	1.12	33.51	0.12	7.95	0.1
	*Paracentrotus lividus*	15.68	1.13	33.56	0.13	7.95	0.11
	*Paracentrotus lividus*	15.78	1.12	33.56	0.1	7.96	0.11
Mn 3 mg/L	*Paracentrotus lividus*	15.67	1.16	33.59	0.14	7.97	0.12
	*Paracentrotus* *lividus*	15.81	1.14	33.56	0.12	7.96	0.12
	*Paracentrotus lividus*	15.84	1.15	33.58	0.1	7.97	0.11
Mn 61.6 mg/L	*Paracentrotus lividus*	15.86	1.15	33.59	0.1	7.95	0.12
	*Paracentrotus lividus*	15.84	1.15	33.59	0.1	7.96	0.11
Main experiment							
	*Paracentrotus lividus*	17.75	0.39	33.48	0.21	8.11	0.06
Controls	*Paracentrotus lividus*	17.69	0.43	33.53	0.24	8.1	0.06
	*Paracentrotus lividus*	17.73	0.35	33.54	0.26	8.11	0.06
	*Paracentrotus lividus*	17.58	0.41	33.52	0.2	8.11	0.06
Mn 1mg/L	*Paracentrotus lividus*	17.67	0.38	33.51	0.16	8.1	0.07
	*Paracentrotus lividus*	17.57	0.42	33.58	0.36	8.11	0.07
	*Paracentrotus lividus*	17.56	0.34	33.62	0.28	8.09	0.08
Mn 3mg/L	*Paracentrotus lividus*	17.43	0.46	33.63	0.32	8.11	0.06
	*Paracentrotus lividus*	17.45	0.42	33.63	0.3	8.11	0.06
	*Echinometra* sp.	25.05	0.54	34.46	0.17	8.1	0.06
Controls	*Echinometra* sp.	25.04	0.52	34.48	0.2	8.1	0.05
	*Echinometra* sp.	25.06	0.51	34.43	0.2	8.11	0.06
	*Echinometra* sp.	25.06	0.51	34.43	0.19	8.11	0.06
Mn 1mg/L	*Echinometra* sp.	25.07	0.52	34.47	0.24	8.1	0.05
	*Echinometra* sp.	25.07	0.52	34.45	0.23	8.09	0.05
Controls	*Prionocidaris baculosa*	25.16	0.37	35.29	0.22	7.96	0.05
	*Prionocidaris baculosa*	25.1	0.37	35.29	0.22	7.96	0.05
Mn 1mg/L	*Prionocidaris baculosa*	25.16	0.36	35.3	0.21	7.95	0.06
	*Prionocidaris baculosa*	25.09	0.36	35.3	0.21	7.95	0.06

**Table 2:** Lengths of spine tips of *Paracentrotus lividus* regenerated during trial experiments (3 spines per individual/ 3 individuals per treatment) and calculated average longitudinal extension rates (ALER) per treatment

	Mn=1 mg/L	Mn=3 mg/L	Mn=61.4 mg/L	Mn=0 mg/L
Spine1	2.581 mm	1.819 mm	0 mm	2.491 mm
Spine2	2.435 mm	2.52 mm	0 mm	2.774 mm
Spine3	2.087 mm	2.687 mm	0 mm	2.756 mm
Spine1	1.65 mm	1.45 mm	0 mm	2.457 mm
Spine2	1.69 mm	2.67 mm	0 mm	2.867 mm
Spine3	1.83 mm	1.61 mm	0 mm	2.894 mm
Spine1	1.118 mm	0.922 mm	0 mm	1.911 mm
Spine2	3.414 mm	3.462 mm	0 mm	2.313 mm
Spine3	2.923 mm	1.386 mm	0 mm	2.648 mm
ALER	183 μm/day	172 μm/day	0 μm/day	214 μm/day

**Table 3:** Lengths of the spine tips of *Echinometra* sp. regenerated during trial experiments (3 spines per individual/3 individuals per treatment) and calculated average longitudinal extension rates (ALER) per treatment

	Mn=3mg/L	Mn=0mg/L
Spine1	0.481 mm	4.742 mm
Spine2	0.053 mm	1.806 mm
Spine3	1.929 mm	1.835 mm
Spine1	1.367 mm	0.083 mm
Spine2	1.894 mm	0.12 mm
Spine3	1.948 mm	0.05 mm
Spine1	2.521 mm	2.387 mm
Spine2	4.656 mm	2.305 mm
Spine3	5.32 mm	2.262 mm
ALER	187 μm/day	144 μm/day
